# Drug Resistance: The Role of Sphingolipid Metabolism

**DOI:** 10.3390/ijms26083716

**Published:** 2025-04-15

**Authors:** Assem Zhakupova, Adelina Zeinolla, Kamilya Kokabi, Shynggys Sergazy, Mohamad Aljofan

**Affiliations:** 1 Department of Biomedical Sciences, Nazarbayev University School of Medicine, Astana 010000, Kazakhstan; a.zhakupova@nu.edu.kz (A.Z.); adelina.zeinolla@nu.edu.kz (A.Z.); kamilya.kokabi@nu.edu.kz (K.K.); 2 Drug Discovery and Development Laboratory, National Laboratory Astana, Astana 010000, Kazakhstan; 3 LLP “VICTUS PHARM”, Astana 010000, Kazakhstan

**Keywords:** sphingolipid metabolism, drug resistance, glucosylceramide synthase, sphingosine kinase, acid ceramidase, sphingosine-1-phosphate, sphingomyelinase

## Abstract

A significant challenge in cancer treatment is the rising problem of drug resistance that reduces the effectiveness of therapeutic strategies. Current knowledge shows that multiple mechanisms play a role in cancer drug resistance. Another mechanism that has gained attention is the alteration in sphingolipid trafficking and the dysregulation of its metabolism, which was reported to cause cancer-associated drug resistance. Sphingolipids are lipids containing sphingosine and have multiple roles, ranging from lipid raft formation, apoptosis, and cell signaling to immune cell trafficking. Recent studies show that in developing cancer cells, altered or dysregulated sphingolipids are associated with drug efflux and promote the survival of cancer cells by bypassing apoptosis. Upregulated levels of the glucosylceramide synthase (GCS), an enzyme that functions in sphingolipid metabolism, lead to the upregulated ABCB1 gene that induces drug efflux from the cancer cells. These bypass mechanisms make drugs that induce apoptosis in tumor cells ineffective. By highlighting the current findings, this review aims to provide a mechanism of drug resistance caused by the dysregulation of glucosylceramide synthase, sphingosine kinase, and acid ceramidase enzymes as possible therapeutic targets to enhance the effectiveness of the currently used chemotherapeutic agents.

## 1. Introduction

Despite advances in treatment strategies, cancer remains a major cause of mortality worldwide [1]. The effectiveness of currently available treatment options is significantly reduced by the emerging drug resistance of cancer cells. Various mechanisms are involved in the development of resistance, including both intrinsic and extrinsic factors [2]. Extrinsic factors include the influence of any external conditions that contribute to the therapeutic resistance, such as the formation of a tumor microenvironment characterized by acidic pH, hypoxia, stromal tissue formation, or immune system modulation [3]. Intrinsic resistance arises as a result of any genetic mutations or adaptations of cancer cells, including epigenetic modification, activated DNA repair mechanisms, bypass of apoptosis by activating survival pathways, and drug efflux [4,5]. Recently, another drug resistance mechanism that involves alterations in sphingolipid metabolism and their trafficking has gained attention [6].

**Figure 1:**
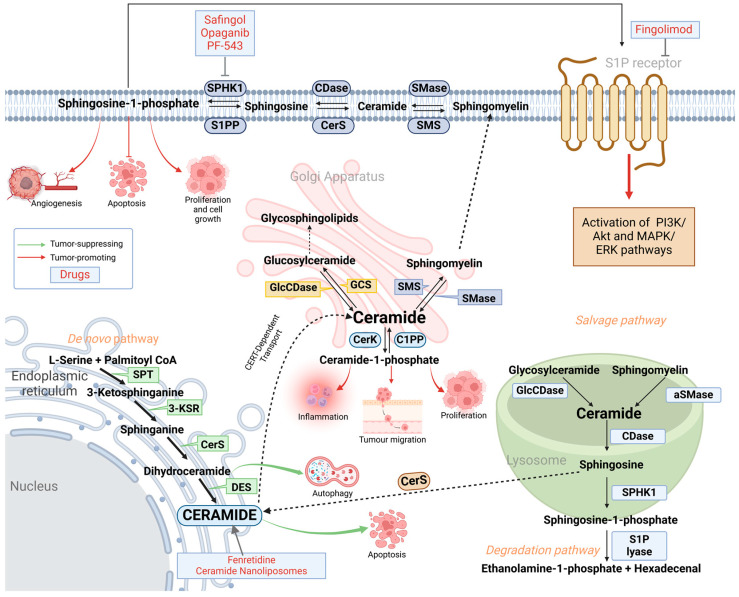
Sphingolipid synthesis pathways. Schematic illustration of sphingolipid synthesis showing that SLs can be formed via de novo synthesis in endoplasmic reticulum through a cascade of enzyme-catalyzed reactions or through the breakdown of glycosylceramides or sphingomyelins in the salvage pathway. Ceramides play a central role in the sphingolipid metabolism and are modified to form more complex sphingolipids. Tumor-suppressing and tumor-promoting functions are indicated with green and red arrows, respectively. Sphingolipids (SLs); GCS, glucosylceramide synthase; SPT, serine palmitoyltransferase; 3-KSR, 3-ketosphinganine reductase; CerS, ceramide synthase; DES, dihydroceramide desaturase; GlcCDase, glucosylceramidase; CERT, ceramide transfer protein; CerK, ceramide kinase; C1PP, ceramide-1-phosphatase; CDase, ceramidase; SMS, sphingomyelin synthase; aSMase, acid sphingomyelinase; SPHK1, sphingosine kinase 1; S1PP, sphingosine-1-phosphatase; SMase, sphingomyelinase.

Sphingolipids (SLs) are lipids with sphingosine backbone that are integrated into the membrane [7]. Sphingosine is an amino alcohol that can be linked to a long fatty acid chain through its amino group, forming a ceramide molecule [8]. Ceramides serve as precursors and are essential for sphingolipid metabolism, which can be either produced in the de novo synthesis or via the recycling of sphingomyelins [9]. Enzymes that participate in the sphingolipid metabolism (Figure 1) and sphingolipids themselves were shown to be significant in several vital cellular functions, including lipid raft formation, cell signaling, apoptosis, vesicle trafficking, cell migration and initiation of immune response [8].

Ceramides were converted into different sphingolipids mediated by the action of enzymes, such as ceramide galactosyltransferase, glucosylceramide synthase or sphingomyelin synthase [10,11]. Alterations in sphingolipid signaling pathways can contribute to the survival of cancer cells and their ability to evade programmed cell death [9]. Furthermore, it was previously reported that in certain cancers, some sphingolipids or their precursors were elevated [12]. Notably, C16:0 and C24:1 ceramides were shown to be increased in patients with pancreatic cancer [13]. Moreover, dysregulation of enzymes linked to sphingolipid metabolism has been shown to promote cancer drug resistance by enhancing cell survival or inhibiting apoptosis, a key mechanism by which cancer cells evade chemotherapeutic treatments [14]. One prominent example is the upregulation of sphingosine kinase, an enzyme associated with increased resistance to chemotherapy in prostate and breast cancers, as well as leukemia [15]. These findings suggest that targeting upregulated enzymes involved in sphingolipid metabolism could potentially address challenges in cancer treatment and medication resistance.

This review focuses on the role of altered sphingolipid metabolism in the progression and emergence of the chemotherapy resistance seen in cancer and its possible mechanism. It also addresses anti-cancer therapeutic strategies and enhancement of existing chemotherapies that regulate sphingolipid metabolism.

## 2. Sphingolipid Metabolism: De Novo, Salvage and Degradation Pathways

The de novo sphingolipid pathway begins in the inner portion of the smooth endoplasmic reticulum (ER), where L-serine and palmitoyl-coenzyme A undergo a condensation reaction to generate 3-ketosphinganine [16]. This reaction is catalyzed by the serine palmitoyltransferase (SPT) enzyme (Figure 1) [17]. 3-ketosphinganine reductase (3-KSR) reduces 3-ketosphinganine to sphinganine, which is then acetylated by ceramide synthase (CerS) to produce dihydroceramide [16]. Finally, ceramide is formed when dihydroceramide is desaturated by the dihydroceramide desaturase (DES) [16]. The resulting ceramide is then translocated to the Golgi apparatus via vesicular or non-vesicular transport mediated by ceramide transfer proteins (CERT) for further processing. This leads to the formation of sphingomyelins and glycosphingolipids catalyzed by sphingomyelin synthase (SMS) and glucosylceramide synthase (GCS), respectively [18]. Figure 1 shows pathways associated with sphingolipid biosynthesis.

Alternatively, ceramides can also be generated by the breakdown of complex sphingolipids within the lysosome, where glucosylceramides and sphingomyelins are converted into ceramides by the enzymes glucosylceramidase (GlcCDase) and acid sphingomyelinase (aSMase), respectively (Figure 1) [18].

Sphingosine can be formed from ceramide by ceramidase enzyme, which is subsequently phosphorylated forming sphingosine-1-phosphate (S1P), a reaction driven by the sphingosine kinase 1 (SPHK1) [6]. Sphingosine-1-phosphate lyase (S1P lyase) can degrade S1P, resulting in the production of ethanolamine-1-phosphate and hexadecenal [19].

Bioactive sphingolipids are involved in the regulation of various cellular functions, such as proliferation, migration, and programmed cell death [20]. Therefore, alterations in their metabolism and expression potentially may lead to cancer progression [7]. Ceramide and S1P have opposite roles in cellular functions, with ceramides promoting cell death (apoptosis) and S1P favoring survival (proliferation, tumor growth, and migration) (Figure 1). Increased conversion of ceramide to S1P, SM or glucosylceramide leads to resistance to existing therapy.

## 3. Enzymes of Sphingolipid Metabolism Involved in Cancer-Related Drug Resistance

Several SL-related enzymes have been linked with resistance to therapy mainly by favoring pro-tumorigenic pathways that lead to cellular proliferation, growth, and migration. Upregulation of several enzymes linked to sphingolipid metabolism has been shown to promote cancer treatment evasion by enhancing cell survival or inhibiting apoptosis, thereby allowing cancer cells to resist existing treatments [14]. This section outlines some of the mechanisms behind the drug resistance.

### 3.1. Glucosylceramide Synthase (GCS) Causes Drug Resistance Through Upregulation of Multidrug Resistance Proteins

Glucosylceramide Synthase (GCS) is an enzyme that catalyzes glycosylation of ceramides by transferring a hexose sugar from UDP-glucose or UDP-galactose, forming glycosphingolipids (GSLs) [21]. GSLs are part of the GSL-enriched microdomains that have many functions, including regulation of the immune responses and apoptosis [22].

The development of multidrug resistance (MDR) in breast and colon cancers has been associated with the overexpression of GCS [23,24]. GCS overexpression can lead to upregulation of MDR proteins, such as P-glycoprotein, which actively effluxes chemotherapeutic drugs out of cancer cells, lowering their intracellular concentration and effectiveness [15]. This was shown by GCS inhibition with RNA interference (RNAi) or siRNA/shRNA that resulted in MDR1 downregulation in doxorubicin-resistant leukemia cells and also increased head and neck cancer (HNC) cells sensitivity to cisplatin [25]. One of the proposed mechanisms involves GCS-mediated modulation of key signaling pathways that regulate the transcription of MDR-related genes since GCS upregulation results in increased production of GSLs that act as signaling platforms. The proposed mechanism involves upregulation of GCS, which promotes phosphorylation of the Akt and Scr that, in turn, leads to the increased level of the β-catenin in the nucleus [26]. β-catenin/Tcf4 (Transcription Factor 4) complex binds to the ABCB1 (ATP binding cassette subfamily B member 1) gene, leading to increased production of the efflux pumps [26].

The trafficking of P-glycoprotein from the Golgi apparatus to the apical membrane is crucial for its function in effluxing drugs and is crucial for its membrane anchoring [27]. Inhibition of GCS has been shown to disrupt this trafficking in HepG2 cells. This disruption leads to the rerouting of newly synthesized GSL analogs to the basolateral membrane instead of their intended apical destination, delaying or reducing the efflux of drugs from the cell [27]. The role of the GSLs in the trafficking of the P-glycoprotein needs to be further elucidated in cancer cells.

Extensive chemotherapy resistance was clinically observed upon treatment with doxorubicin. It acts as an intercalating agent and disrupts the topoisomerase-II-mediated DNA repair. Unresponsive colon and breast cancer samples showed that upon doxorubicin treatment, the levels of GCS expression significantly increased [26]. Moreover, in head and neck cancers, inhibition of GCS has been shown to enhance the sensitivity of cancer cells to cisplatin treatment [28]. A recent study also showed that targeting GCS significantly enhanced the proapoptotic effects of osimertinib in the non-small cell lung cancer cell lines resistant to the drug [12].

### 3.2. Dysregulation of Sphingosine Kinase (SPHK) and Sphingosine-1-Phosphate (S1P) Promotes Drug Resistance Through Activation of Pro-Tumorigenic Pathways

Sphingosine kinases belong to the diacylglycerol kinase family and phosphorylate sphingosine to produce sphingosine-1-phosphate (S1P) [29]. Healthy levels of S1P are maintained through the ceramide-S1P rheostat. In the case of any imbalance in the level of S1P, S1P lyase degrades excessive S1P via a degradation pathway, or it can be dephosphorylated by the sphingosine-1-phosphate phosphatase (S1PP) [29]. Any imbalance in this regulatory mechanism may lead to the elevated expression of SPHK enzymes or S1P. S1P exhibits pro-tumorigenic functions, including inhibition of apoptosis, promotion of cell growth, differentiation, cell migration and angiogenesis [30]. Therefore, overexpressed levels of SPHK enzymes have been associated with several tumors, including squamous cell and hepatocellular carcinomas as well as breast, prostate, bladder, colorectal and ovarian cancers [31].

**Figure 2:**
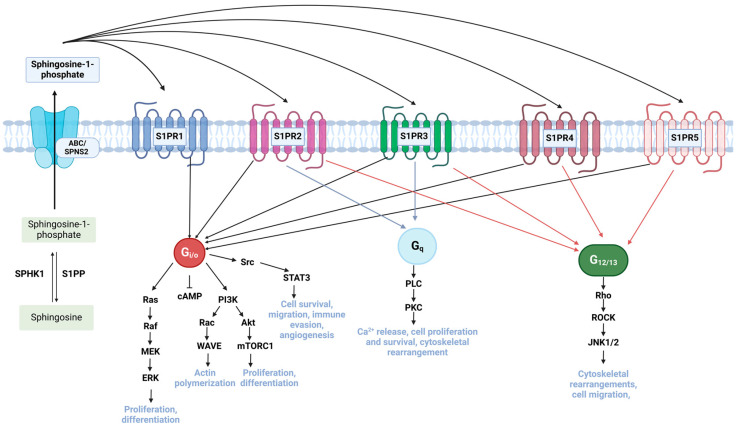
S1P-mediated signaling pathways. Schematic illustration showing intracellular S1P can be transported via ABC/SPNS2 transporters and bind S1PR1-S1PR5 G-protein coupled receptors and activate several pro-tumorigenic pathways that lead to cellular proliferation, growth and migration. Gi/o activates pro-survival pathways PI3K/Akt or Ras/MAPK/ERK, which leads to increased cell proliferation and growth of the cancer cells, making existing apoptosis-inducing treatment inefficient [31]. S1P can upregulate the production of P-glycoprotein, which actively extrudes cancer-targeted drugs out of the cell, thereby enhancing tumor drug resistance [29]. SPHK activates the JAK/STAT signaling pathway via the S1PR1 receptor in colon cancer cell lines, which induces migration and invasiveness [32].

Intracellular S1P is exported from the cell via SPNS2 or ABC transporter (Figure 2) [32]. Next, S1P binds and activates G protein-coupled receptors S1PR1-S1PR5 that are associated with different heterotrimeric G protein alpha subunits, including Gi/o, G12/13, and Gq [33]. Particularly, Gi/o activates pro-survival pathways PI3K/Akt or Ras/MAPK/ERK that lead to the increased cell proliferation and growth of the cancer cells, making chemotherapeutic drugs causing apoptosis inefficient [34]. Figure 2 summarizes S1P-mediated signaling pathways.

It was demonstrated that S1P can also upregulate the production of P-glycoprotein, which actively extrudes cancer-targeted drugs out of the cell, thereby enhancing the ability of tumor cells to evade chemotherapy [32]. SPHK also activates the JAK/STAT signaling pathway via S1PR1 receptor activation in colon cancer cell lines that promotes their migration and invasiveness (Figure 2) [35].

In prostate cancer cell lines, targeting SPHK1 and inhibiting its activity improved the efficacy of camptothecin and docetaxel [36]. A more recent study also demonstrated that doxorubicin-resistant breast cancer cells are more susceptible to doxorubicin if SPHK1 is inhibited with fingolimod, thereby enhancing its therapeutic effect [37].

In chronic myeloid leukemia (CML), an abnormally high expression of SPHK1 was shown to exacerbate the resistance to the imatinib drug. The proposed mechanism of drug resistance is through the upregulated SPHK1, which suppresses the expression of protein phosphatase 2 (PP2A). This inhibits the proteosomal degradation of the mutated tyrosine kinase receptor Bcr-Abl1, which confers further drug resistance and reduces the responsiveness of cancer cells to drugs [38]. Another study also showed that CML cells can develop resistance to imatinib through the activation of PI3K/Akt and mTOR signaling pathways that improve cellular proliferation and invasiveness (Figure 2) [39].

### 3.3. Abnormal Acid Ceramidase (AC) Levels Promote Drug Efflux in Cancer Cells

Acid ceramidase is an enzyme that facilitates the breakdown of ceramide into sphingosine and functions optimally at an acidic pH of 4.0–4.5, being localized in the lysosome [40]. Since sphingosine can be further converted into S1P, which promotes cell survival, targeting AC has emerged as a potential therapeutic strategy to inhibit cancer cell proliferation through the Akt or MAPK pathways and induce apoptosis by ceramide accumulation [41]. AC promotes cell survival not only through NF-κB activation but also via modulation of the Akt/mTOR and Bcl-2 family signaling pathways, further reinforcing apoptosis resistance and enhancing tumor progression.

Abnormal AC levels were associated with prostate cancer, acute myeloid leukemia (AML), and head and neck cancer [42,43]. Increased levels of acid ceramidase (AC) enable cancer cells to bypass apoptosis, thereby contributing to drug resistance. It was previously shown that AC expression increases upon radiation in prostate cancer and enhances the resistance to radiation, thus resulting in the relapse of cancer [44]. Prostate cancer cells became more sensitive to radiation when the expression of AC was inhibited using siRNA [44]. Moreover, upregulated AC levels in prostate cancer made cells unresponsive upon treatment with doxorubicin, etoposide and cisplatin, further underscoring its role in chemoresistance [15].

Recent studies have demonstrated that in AML, abnormally high AC levels were associated with P-glycoprotein upregulation and increased drug efflux, a key mechanism of MDR [41,45]. Interestingly, overexpressed AC increases the activity of the NF-κB signaling pathway, which in turn upregulates P-glycoprotein expression. Inhibiting NF-κB signaling results in the decrease of P-glycoprotein expression levels, suggesting that AC may cause drug resistance through the NF-Κb-dependent pathway [41]. Additionally, beyond its role in NF-κB signaling, AC influences multiple apoptotic and survival pathways, including those involved in autophagy regulation [42]. AC modulates the ceramide-S1P balance, affecting lysosomal stability, mitophagy, and overall tumor cell survival [19,42,46]. Accumulated data suggest that autophagy plays a dual role in cancer, acting as both a tumor suppressor and a survival mechanism depending on the cellular context. AC, by regulating the ceramide-S1P balance, may influence autophagy in a manner that enhances cancer cell survival. Increased AC expression has been linked to reduced autophagic stress and sustained mitophagy, which collectively contribute to chemoresistance. This highlights a potential therapeutic avenue where targeting AC, in combination with autophagy regulators, may help overcome drug resistance in cancer cells.

### 3.4. Downregulation of Sphingomyelinases (SMase) Mediates Apoptosis Resistance

Sphingomyelinases (SMases) are enzymes that catalyze the breakdown of sphingomyelin (SM) to generate ceramide [15,47]. Within the cell, five distinct SMase subtypes exist, namely acidic zinc-dependent and zinc-independent SMases, neutral magnesium-dependent and magnesium-independent SMases and basic SMases [48]. Each subtype exhibits differential regulation and activity under varying cellular conditions, influencing cancer cell fate.

Upregulation of SMases was shown to be associated with several malignancies, including glioblastoma, colon and ovarian cancers and non-small cell lung cancer (NSCLC), conferring resistance against both chemotherapy and radiotherapy [15].Overexpression of acidic sphingomyelinases aSMase in glioblastoma cells leads to higher sensitivity to gemcitabine and doxorubicin therapy, due to elevated ceramide levels promoting apoptosis [49]. However, in other contexts, such as lung cancer, high aSMase expression has been associated with resistance to cell death, potentially through compensatory mechanisms involving other SMase subtypes. Moreover, in lymphatic cancer cell lines, high levels of SMs induced CD95-mediated apoptosis, while in lung and breast cancer cells, it causes cytochrome c release, leading to apoptosis [50]. It was found that increased amounts of acid SMase resulted in elevated ceramide levels, which causes apoptosis [50]. However, the interplay between SMases and AC introduces an additional layer of complexity in apoptosis regulation with differing p53 status. Interestingly, in glioblastoma cancer cell lines with deficient p53 pathway, upregulation of SM and ceramide causes apoptosis. In contrast, in cells with wild-type p53 status, p53 leads to a decrease in ceramide through upregulation of AC, thereby promoting apoptosis evasion [50]. This highlights a crucial adaptive mechanism by which cancer cells modulate ceramide metabolism to resist cell death.

## 4. Clinical Implications

Inhibitors of enzymes associated with sphingolipid metabolism were designed and employed both in preclinical and clinical studies to resolve the chemotherapy tolerance of several cancers, which are listed in Table 1.

### 4.1. Targeting S1P Signalling Pathway

Inhibition of S1P formation and S1P-associated signaling is the most promising target for suppressing cancer formation, since it regulates several pro-tumorigenic pathways. One prominent example is SK1-I/II (BML-258), a selective competitive inhibitor of SPHK1/2, which was shown to be successful in several preclinical studies for gastric cancer, colorectal cancer, and AML [58,60,80]. Moreover, monoclonal antibodies against S1P called sphingomab (sonepcizumab) are currently in the Phase II clinical trial for metastatic renal cell carcinoma and sunitinib-resistant renal cell carcinoma treatment [64].

Opaganib (ABC294640) also underwent a Phase I clinical trial for advanced solid tumors and is under Phase II clinical trial for hepatocellular carcinoma treatment [35,67,68]. It acts as a selective inhibitor of SPHK2, limiting the formation of S1P and inhibits tumor cell survival, proliferation, angiogenesis, and inflammation [81] (Figure 1). Fingolimod (FTY720) conversely acts on the S1PR1 receptor and directly internalizes and degrades it, thereby preventing S1P signaling activation (Figure 1) [82]. Currently, this drug is already approved to treat relapsing–remitting multiple sclerosis [83]. The mechanism of action of fingolimod makes it a promising candidate for treating tumors associated with chronic inflammation [84]. Moreover, in several preclinical studies, fingolimod was shown to be successful in initiating apoptosis in prostate, breast, lung, ovarian, and colorectal cancers [51,52,85]. One of the proposed mechanisms through which fingolimod exerts its action is by triggering the formation of large ceramide-enriched membrane structures known as ceramidosomes. Ceramidosomes are composed of the ceramide–myosin IIA–RIPK1 complex, which triggers necroptosis and inhibits cancer progression [86].

### 4.2. Induction of Apoptosis in Cancer Cells by Ceramide Nanoliposomes

Ceramide induction is a potent target in treating cancer cells due to its several protective roles, particularly its ability to initiate apoptosis. Ceramide has poor pharmacokinetic parameters due to rapid metabolism and poor solubility and may be toxic to normal cells [87]. To ensure more targeted delivery and to limit its nonspecific interactions with healthy cells, ceramide is encapsulated within nanoliposomes. Several studies have demonstrated C6-ceramide nanoliposome efficiency in cancer treatment. For example, treatment with C6-ceramide nanoliposome inhibits melanoma metastasis by reducing integrin affinity and suppresses cancer cell migration [88]. Another study has shown that a nanoliposome-loaded C6-ceramide induced immune response against cancer in the mice model with a liver tumor that resembles human hepatocellular carcinoma, showing a promising strategy in immunotherapy [11]. Ceramide nanoliposomes are also being investigated in phase I clinical trials for patients with advanced solid tumors, where increased safety profiles and potential efficacy were demonstrated [69].

### 4.3. Natural Compounds Targeting Abnormal Sphingolipid Expression in Cancer Therapy

Recent advances in cancer research highlight the potential of natural compounds in modulating sphingolipid metabolism that can possibly enhance existing treatment methods. Many of them act by upregulating the levels of ceramide either by targeting ceramide-metabolizing enzymes or causing its overproduction. One such compound is sanguirine (SNG), an alkaloid extracted from the Sanguinaria canadensis plant [89]. Mechanistically, SNG was shown to induce hydrogen peroxide-dependent ceramide generation in DU145 human prostate cancer cells [90]. Moreover, SNG leads to ceramide accumulation by inhibiting ceramide-metabolizing enzymes such as CDase and GCS [90]. Since elevated ceramide is known for its role in activating the apoptotic cascade, SNG can be potentially used in a combination therapy by sensitizing resistant cancer cells to apoptosis.

Another example includes SPHK1 inhibitors F-12509A produced by the Trichopeziella barbata fungi and B-5354C produced by marine bacterium [91]. F-12509A is a competitive inhibitor of SPHK1 that leads to ceramide accumulation and reduces S1P levels, while B-5354C inhibits SPHK1 non-competitively by binding to regulatory domains rather than the S1P-binding domain [92]. Further research is needed to discover the effectiveness of those naturally derived SPHK1 inhibitors. An alternative inhibitor of SPHK of natural origin is tricin, which is contained in many Monocotyledons [93]. Unlike F-12509A or B-5354C, tricin acts on SPHK indirectly by decreasing the phosphorylation of Protein Kinase C alpha (PKCα), thereby preventing SPHK activation by PKCα. This was confirmed in a non-small cell lung cancer (NSCLC) cell line, showing that tricin suppresses PKCα/SPHK/S1P signaling [93].

Another compound, Englerin A, was extracted from the Phyllanthus engleri plant [94]. Interestingly, it has been shown to be highly effective in inhibiting the growth of renal cell carcinoma cell lines [95]. The proposed mechanism indicates that Englerin A induces apoptosis in cancer cell lines by upregulating the levels of ceramide, probably by activating the sphingomyelinase enzyme that converts sphingomyelin into ceramide [95].

Jaspine B, a compound first isolated as a cytotoxic component of the marine sponge Pachastrissa sp, gained attention for its potential anticancer properties [96]. The antiproliferative effect of Jaspine B can be explained by the increase in intracellular ceramide levels that induces apoptosis. In studies with the HeLa cell line, Jaspine B treatment resulted in elevated ceramide levels, which correlated with an increase in aSMase enzyme expression, responsible for converting sphingomyelin into ceramide [97].

One study conducted in the HeLa cell line showed that increased ceramide levels are associated with increased expression of aSMase enzyme, which catalyzes the conversion of sphingomyelin into ceramide upon treatment with Jaspine B [97].

Lastly, curcumin, a polyphenol derived from Curcuma longa, may also exert anticancer effects due to its ability to increase intracellular ceramide levels. In one study that focused on an in vitro model of impaired intracellular lipid trafficking in glial cells, curcumin increased the biosynthesis of both dihydroceramide and ceramide [98]. Additional research is needed to confirm whether curcumin has a similar effect in cancer models.

Together, these natural compounds may exert a therapeutic potential in targeting sphingolipid metabolism in cancer treatment. Further investigation and clinical exploration is needed to describe their efficacy and safety profiles.

## 5. Discussion

Drug resistance poses a significant challenge in cancer therapy, thereby reducing the effectiveness of treatment strategies. There are a number of reported mechanisms that might explain the ability of cancer cells to evade treatments, such as enhancement in efflux pumps and DNA damage repair, apoptosis inhibition, epigenetic modifications, and epithelial-mesenchymal transition. All of these processes contribute to increased cancer cell resistance and invasiveness. Recently, sphingolipids appeared to play a role in cancer-associated drug resistance. Sphingolipids are bioactive molecules that play important roles in apoptosis, cellular metabolism, signaling and trafficking [20]. Disruption of lipid trafficking and its metabolic dysregulation results in alteration of sphingolipids levels. This, in turn, leads to drug efflux and cancer cell survival by evading apoptosis and causing cancer-associated drug resistance. Bypass mechanisms used by tumor cells make current treatment ineffective. Several inhibitors or activators exist that target enzymes of sphingolipid metabolism and have reported successful enhancement of chemotherapy-associated drug resistance.

Disturbances in sphingolipid metabolism-associated enzymes and resulting metabolites may lead to cancer-associated evasion of chemotherapy. Dysregulation in the expression of SL metabolism enzymes, such as glucosylceramide synthase (GCS), sphingosine kinase and acid ceramidase, leads to cancer cell survival and resistance through drug efflux, bypassing apoptosis or activation of pro-tumorigenic pathways. This suggests that enzymes of sphingolipid synthesis can be used as therapeutic targets in overcoming tumor drug resistance. Since S1P signaling can lead to the activation of cell survival, proliferation and migration, it serves as the most promising target for cancer treatment. Several preclinical and clinical studies are being conducted to study S1P signaling inhibitors. Overall, elucidating potential drugs that target specific sphingolipids and their roles can significantly resolve tumor-associated resistance to treatment.

A promising method of overcoming sphingolipid-associated evasion of chemotherapy in cancer is the application of combination therapies, where sphingolipid-targeted inhibitors are coupled with chemotherapy to reduce the invasiveness, proliferation, and survival of cancer cells. For example, preclinical testing of co-delivery of paclitaxel (used for treatment of advanced carcinoma of the ovary) with DES inhibitor fenretinide showed significant enhancement of their synergistic anticancer effects, reduced toxicity, and improved pharmacokinetic properties compared to free drugs [71]. Another study evaluated the combination of fenretinide (4-HPR) and venetoclax (ABT-199) in preclinical models of recurrent high-risk neuroblastoma [99]. The results showed that the combination was highly synergistic in neuroblastoma cell lines and patient-derived xenografts with high BCL-2 expression, significantly improving event-free survival in mice [99]. Moreover, a combination of SK-II and paclitaxel significantly increased the paclitaxel sensitivity of lung cancer cell lines, suggesting a possible therapeutic approach in targeting NSCLC [100].

Despite the demonstrated synergistic anticancer effects, these inhibitors can present significant challenges, including dose-limiting toxicities and off-target effects that can complicate their transition to clinical use. For instance, the combination of safingol with cisplatin, although effective in treating advanced solid tumors, was associated with hepatic toxicity, as evidenced by elevated liver enzymes in patients, leading to their withdrawal from clinical trials [101]. Moreover, opaganib (ABC294640) in Phase I clinical trials showed adverse drug toxicities, including nausea, vomiting and neurological disorders [68]. No clinical trials for SK-II and Fingolimod were conducted. Fingolimod itself has many side effects, including suppression of migration and activation of CD8+ and CD4+ T cells, which prevents effective tumor cell infiltration and killing [102,103]. While combination therapies involving sphingolipid-targeted inhibitors and chemotherapy show promise in enhancing anticancer effects and overcoming drug resistance, challenges such as dose-limiting toxicities and off-target effects must be addressed before these treatments can be widely applied in clinical settings.

Moreover, studies have shown that targeting sphingolipids in cancer treatment can be very complicated due to heterogeneous sphingolipid profiles [104]. This significantly affects the efficacy of the current sphingolipid enzyme inhibitors, which makes it necessary to implement personalized approaches. Additionally, several parameters of the sphingolipid inhibitors, like low solubility, low cell permeability, and lack of specificity, must be addressed [105]. More advanced delivery systems, like in ceramide nanoliposomes mentioned earlier, can be explored.

Despite the fact that many drugs targeting sphingolipid metabolism are being investigated in clinical trials, not all compounds showed expected and desired efficacy against cancer. For example, to reduce off-target effects, selective inhibitors of SPHK enzyme isoforms, including opaganib, were developed. Interestingly, SPHK1 and SPHK2 can compensate for each other’s deficiencies, showing redundancy in function, which reduces the efficiency of anticancer therapy [106]. Therefore, further clinical studies need to be conducted to determine the effective and safe combination therapies for various cancer types.

## Figures and Tables

**Table 1:** List of drugs and inhibitors targeting sphingolipid metabolism-associated enzymes.

Name	Mechanism of Action	Cancer Type	Status	References
Fingolimod (FTY720)	Inhibits S1P signaling by acting as a functional antagonist of S1PR1	Prostate cancer	Preclinical studies	[51]
		Breast cancer	Preclinical studies	[52,53,54]
		Pancreatic cancer	Preclinical studies	[55]
		Thyroid cancer	Preclinical studies	[56]
		Colorectal cancer	Preclinical studies	[57,58]
JTE013	Selectively inhibits S1PR2		Preclinical studies	[59]
SK1-I/II (BML-258)	Competitive SPHK1/2 inhibitor	Gastric cancer	Preclinical studies	[60]
		Glioblastoma	Preclinical studies	[58]
		Breast cancer	Preclinical studies	[61]
		Colorectal cancer	Preclinical studies	[58]
Safingol	SPHK1 inhibitor	Breast and Colon cancer	Preclinical studies	[62]
		Multiple Myeloma	Preclinical studies	[63]
Sphingomab (sonepcizumab)	Monoclonal antibody neutralizing S1P	Metastatic renal cell carcinoma	Phase II clinical trial	[64]
Opaganib (ABC294640)	Selectively inhibits SPHK2	Breast cancer	Phase I clinical trial for advanced solid tumors. Phase II clinical trial for hepatocellular carcinoma. Preclinical studies for triple negative breast cancer and granular lymphocyte leukemia.	[61,65,66]
		Granular lymphocyte leukemia	Preclinical studies	[67]
		Advanced solid tumors	Phase I clinical trial	[68]
Ceramide nanoliposomes	Ceramide inducer	Advanced solid tumors.	Phase I clinical trials	[69]
Fenretinide	DES inhibitor	Lung and colorectal cancer	Preclinical studies	[70]
		Ovarian and breast cancer	Preclinical studies	[71]
		AML	Preclinical studies	[72,73]
		Solid tumours and lymphoma	Phase I clinical trial	[74]
		Ascitic Ovarian Cancer	Phase I-II clinical trial	[75]
		Breast cancer	Phase II clinical trial	[76]
		Bladder cancer	Phase II clinical trial	[77]
LCL521	Acid ceramidase inhibitor	Head and neck cancer	Preclinical studies	[78]
Carmoflur	Acid ceramidase inhibitor	Glioblastoma	Preclinical studies	[79]
